# Maternal Diet Quality during Pregnancy Is Associated with Neonatal Brain White Matter Development

**DOI:** 10.3390/nu15245114

**Published:** 2023-12-15

**Authors:** Xiaoxu Na, Charles M. Glasier, Aline Andres, Xiawei Ou

**Affiliations:** 1 Department of Radiology, University of Arkansas for Medical Sciences, Little Rock, AR 72205, USA; 2 Arkansas Children’s Nutrition Center, Little Rock, AR 72202, USA; 3 Arkansas Children’s Research Institute, Little Rock, AR 72205, USA; 4 Department of Pediatrics, University of Arkansas for Medical Sciences, Little Rock, AR 72205, USA

**Keywords:** maternal diet during pregnancy, Healthy Eating Index (HEI), neonatal brain development, diffusion tensor imaging (DTI)

## Abstract

Maternal diet and nutrient intake are important for fetal growth and development. In this study, we aim to evaluate whether there are associations between maternal diet quality and the offspring’s brain white matter development. Healthy pregnant women’s (N = 44) nutrition intake was assessed by the Healthy Eating Index-2015 (HEI-2015) during the first, second, and third trimesters, respectively. Correlations between MRI diffusion tensor imaging measured fractional anisotropy (FA) of the neonatal brain and the HEI-2015 scores were evaluated using voxel-wise analysis with appropriate multiple comparisons correction and post hoc analysis based on regions of interest. Significant correlations were found between sodium scores at the first trimester of pregnancy and mean neonatal FA values in parietal white matter (R = 0.39, *p* = 0.01), anterior corona radiata (R = 0.43, *p* = 0.006), posterior limb of internal capsule (R = 0.53, *p* < 0.001), external capsule (R = 0.44, *p* = 0.004), and temporal white matter (R = 0.50, *p* = 0.001) of the left hemisphere. No other correlations were identified. In conclusion, the relationships between the maternal sodium intake score and the neonatal white matter microstructural development indicate sodium intake patterns better aligned with the Dietary Guidelines for Americans during early pregnancy are associated with greater white matter development in the offspring’s brain.

## 1. Introduction

While undernutrition is detrimental to human health, it is also well known that excessive intake of calories and certain nutrients such as saturated fats, added sugars, and sodium are also health concerns, which can be associated with chronic health conditions including obesity, cardiovascular diseases, type 2 diabetes, certain cancers, and bone health disorders [1]. On the other hand, nutrient-rich and balanced dietary patterns can help to maintain good health throughout every stage of life [2]. When it comes to pregnancy, healthy nutrition helps pregnant women handle the demands on both their own bodies and the growing fetuses. Inadequate levels of key nutrients during fetal development may lead to altered programming within fetal tissues and contribute to unfavorable long-term outcomes [3]. There is no doubt that a balanced and nutrient-rich diet during pregnancy is essential for optimal fetal health [4]. 

Of special interest is that maternal dietary quality and nutrient intake during pregnancy have been found to be associated with offspring cognition and behavior development during childhood. A recent study showed higher maternal dietary intake quality during pregnancy was associated with better visual-spatial skills [5] and cognitive scores [6] in offspring at early childhood, in addition to the literature reports of positive associations with higher intelligence and executive functions at mid-childhood [5,7]. Meanwhile, there is also evidence showing associations between specific components of maternal dietary intake and the cognitive, neurophysiological, and behavioral outcomes of offspring. For example, higher maternal seafood intake was reported to be associated with an increase in the offspring’s intelligence quotient (IQ) [8,9]. Higher fruit consumption during pregnancy was reported to be positively associated with 1-year cognitive development [10]. Higher maternal fruit and vegetable consumption was associated with higher verbal, performance, and IQ of 8-year children [11]. Higher nut intake during early pregnancy was reported to be associated with greater long-term child neuropsychological development [12]. On the other hand, one study showed that higher maternal starch intake was reported to be inversely associated with child performance IQ [13]. Another study reported that higher maternal sucrose consumption was inversely associated with mid-childhood Kaufman Brief Intelligence Test (KBIT-II) non-verbal scores, while higher maternal sugar-sweetened beverage consumption was reported to be inversely associated with mid-childhood cognition [14].

Brain development during early age may be the driving factor of neurodevelopmental outcomes in later childhood. For example, both macrostructural and microstructural growth in the brain were associated with cognitive performance in children [15,16]. Nutrition and specific nutrient intake during pregnancy may directly affect fetal brain development, which could lead to neurodevelopmental consequences in later life. This impact may be assessed by neuroimaging during or soon after birth, using techniques such as diffusion tensor imaging (DTI), which is a non-invasive magnetic resonance imaging (MRI) technique that is very sensitive to brain white matter (WM) microstructural integrity [17]. This is also the rationale for our study. WM development is essential for neurological functioning and has important implications for neurodevelopmental outcomes. DTI has been widely used in studies evaluating the normal and abnormal development of WM microstructures and their relationship with cognitive functions [18,19,20]. 

Our hypothesis for this study was that maternal dietary quality during pregnancy would impact fetal brain WM microstructural development, which can be seen using neonatal brain imaging such as DTI. We assessed healthy pregnancy women’s diets at each trimester of pregnancy and evaluated the alignment of their dietary intake with a Healthy Eating Index (HEI) defined by the Dietary Guidelines for Americans (DGA). We also performed a brain MRI examination of their newborns at ~2 weeks of age. The neonatal WM microstructural development evaluated by DTI and its correlation with maternal HEI during pregnancy was studied and reported.

## 2. Materials and Methods

### 2.1. Subjects

All study procedures were approved by the Institutional Review Board at the University of Arkansas for Medical Sciences. Written informed consent was obtained from all participants (pregnant women) for themselves and their newborns being included in the study. The study cohort is part of a large longitudinal study (The Glowing Study, clinicaltrials.gov identifier NCT01131117) at the Arkansas Children’s Nutrition Center. All pregnant women without medical complications who were recruited at <10 weeks of gestation to the study met these criteria: second parity, singleton pregnancy, ≥21 years of age, and conceived without assisted fertility treatments. Those with pre-existing medical conditions such as diabetes mellitus, seizure disorder, serious psychiatric disorders, drug or alcohol use during pregnancy, sexually transmitted diseases, and medical complications developed during pregnancy, such as gestational diabetes and pre-eclampsia, were excluded. For neonates, those born preterm (<37 weeks of gestation) or with medical conditions or medications known to influence fetal growth and development were also excluded. All newborns were healthy at birth, with an Apgar score >8, and without birth complications. The study cohort was originally designed to study the impact of maternal obesity during pregnancy on offspring brain development. In total, 46 pregnant women were recruited from the larger cohort, and 44 pregnant women and newborn dyads completed both the HEI assessments and the DTI scan and were included in this study.

### 2.2. HEI Assessment of Pregnant Women

Dietary intakes during pregnancy were assessed using 3-day food records using self-documentation and report. Participants were instructed to record all food, beverage, medication, and supplement intake they ate throughout each day for 2 weekdays and 1 weekend day. Participants were provided with a booklet to evaluate the size or volume of intake for each component of their meal, including drinks, medications, or solid foods on the food records. The records were reviewed by a trained research assistant to clarify any information and query any additional intake that may have been missed. The dietary data were then analyzed using the Nutrition Data System for Research software 2018 (Nutrition Coordinating Center, University of Minnesota, Minneapolis, MN, USA) [21]. Estimation of dietary intake was calculated at each trimester, and the Healthy Eating Index-2015 (HEI-2015) was derived from published formulas [22,23]. HEI-2015 is a measurement for assessing dietary quality, specifically the degree to which the dietary intake aligns with the recommendations in the 2015–2020 Dietary Guidelines for Americans (DGA 2015–2020). HEI-2015 has been used in a variety of research settings involving a range of calorie intake levels, such as to monitor the diet quality of the U.S. population, to study the associations between diet quality and health outcomes, to examine the food supply, and to adapt to global settings according to the Food and Nutrition Service of the USDA. Numerous research studies have been using HEI metrics to assess diet quality [24], and the psychometric properties of HEI, including content and construct validity and reliability in a large study cohort [22] and in pregnant women [25] have been evaluated. The HEI-2015 carries 13 components and a total/composite score. Briefly, each component is scored on a density basis out of 1000 calories, with the exception of fatty acids, which is the ratio of unsaturated to saturated fatty acids. The components of total fruits, whole fruits, total vegetables, greens and beans, total protein foods, seafood, and plant proteins are scored on a range of [0, 5]. The components of whole grains, dairy, fatty acids, refined grains, sodium, added sugars, and saturated fats are scored on a range of [0, 10] (Table 1). The total score is a summed-up with a maximum value of 100. The scoring standard for each component [23] is also presented in Table 1. Among all the components, refined grains, sodium, added sugars, and saturated fats are moderation components representing dietary elements for which there are recommended limits to consumption (i.e., higher scores reflect lower intakes and better alignment with the DGA 2015–2020). The rest of the components are adequacy components representing dietary elements that are encouraged (i.e., higher scores are a reflection of higher intakes and better alignment with the DGA 2015–2020). Overall, a higher total HEI-2015 score indicates a diet that aligns better with the DGA 2015–2020. Among all the 44 mother–newborn dyads, 43 provided food records that were scored (using HEI-2015) in the first trimester, and all 44 provided food records that were scored (using HEI-2015) in the second and the third trimester of gestation.

### 2.3. MRI Data Acquisition

All newborns underwent an MRI examination at ~2 weeks of age at Arkansas Children’s Hospital Department of Radiology. DTI imaging data were acquired using a 1.5T Philips scanner (Philips, Best, The Netherlands) with an 8-channel SENSE head coil during natural sleep without sedation. A neonatal brain MRI protocol including regular diffusion, susceptibility-weighted, and 3D T1-weighted imaging was used to screen for incidental findings that may need medical attention. In addition, a single-shot spin echo EPI sequence with TR/TE 4200 ms/66 ms and 180 mm × 180 mm field of view, 90 × 90 acquisition matrix, and 3 mm slice thickness (30–36 continuous axial slices for each brain scan) and diffusion-weighting gradients in 15 uniformly distributed directions with a b-value of 700 s/mm^2^ was used to acquire DTI data in a very short scan time. 

### 2.4. MRI Data Analysis

MRI data were sent to the institution’s picture archiving and communication system (PACS) after the scan to be screened by a neuroradiologist to exclude abnormalities needing medical attention. DTI data was exported to a desktop workstation with FSL 6.0.4 (Analysis Group, the Oxford Centre for Functional MRI of the Brain, Oxford, UK) installed on a VMware Linux virtual machine 15 Player (Broadcom Inc., Palo Alto, CA, USA) for pre-processing. Brain extraction, eddy currents, and movement corrections were performed, and eigenvalues for the diffusion tensors were then computed, and DTI parameter maps were generated using the FDT DTIFIT toolbox in FSL. Tract-based spatial statistics (TBSS) methods were used for DTI data analysis [26]. In short, the fractional anisotropy (FA, a main DTI parameter sensitive to white matter microstructural integrity with great contrast between white and gray matters) maps were processed and aligned to each other in order to identify the most representative one (the target) that was determined as requiring the least amount of total warping. This determined target consequently served as a neonatal template for nonlinear registration. All FA maps were then registered to this template and were skeletonized to illustrate their major white matter tracts (defined as FA ≥ 0.1, which was adjusted from the adult threshold to reflect lower FA values in newborns). These processed FA maps were then used for voxel-wise statistical analysis. 

### 2.5. Statistics

In order to examine the associations between HEI-2015 scores and newborns’ brain WM FA values, the randomization program in FSL was used to perform voxel-wise correlation analysis between FA values in the neonatal brain WM and all components of HEI-2015 assessed at each trimester of pregnancy, respectively. Randomization tests were performed with the threshold-free cluster enhancement (TFCE) option and 5000 permutations for both positive and negative correlations between the FA and each HEI-2015 score. To control for potential confounders, each newborn’s sex and postmenstrual age at MRI were included in the permutation as covariates [27]. Since the study cohort was originally designed to study the impact of maternal obesity during pregnancy on offspring brain development, maternal BMI was also included as a covariate due to the reported effects [28,29]. Other potential confounders during pregnancy or around birth were not included due to limited sample size and statistical power. Correlations were evaluated using voxel-wise non-parametric tests corrected for multiple comparisons using the family-wise error rate (FWE). Clusters identified with FWE-corrected two-tailed *p* ≤ 0.05 in the voxel-wise analyses and with a size bigger than 40 voxel size were further tested for correlations using regions of interest (ROIs) analyses. Each WM ROI included one cluster, and the mean FA values for each ROI were extracted for each subject. Post hoc analysis of partial Spearman’s rank correlation test using Matlab software 2018b (Mathworks Inc., Natick, MA, USA) with the same covariates controlled was performed to confirm significant relationships between mean FA values in each ROI and the HEI component scores. Correlation coefficients (R values) were calculated, and *p* ≤ 0.05 was regarded as significant and reported.

## 3. Results

**Figure 1:**
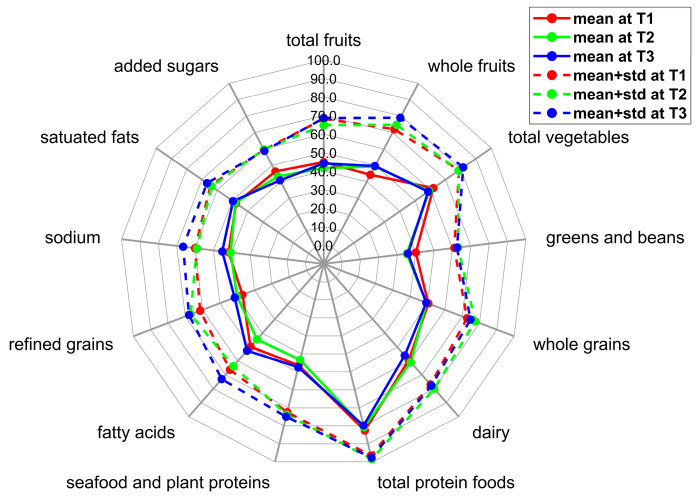
HEI-2015 scores at each trimester of pregnancy were plotted with mean (solid lines) and one standard deviation from the mean (dashed lines) to show their alignment with the DGA 2015–2020. The outer edge of the wheel represents a maximum score in theory and perfect alignment with the DGA 2015–2020. Inner circles represent lower percentages and lower alignment with the DGA 2015–2020.

Table 2 summarizes the demographic information for the mother–newborn dyads involved in the study. All components of the HEI-2015 scores at different time points throughout the pregnancy are shown in Table 3. ANOVA tests between time points were performed for each HEI component. The total HEI-2015 score showed consistency throughout the entire pregnancy, and there were no significant differences identified for each HEI parameter between any two time points. A radar plot displaying the extent to which each component is aligned with DGA 2015–2020 is shown in Figure 1. In our study, intake of total protein foods was the most aligned (~80% during all trimesters) with the DGA 2015–2020, while total fruit, greens and beans, refined grains, sodium, and added sugar were not well-aligned with the DGA 2015–2020 (as low as ~40%).

**Figure 2:**
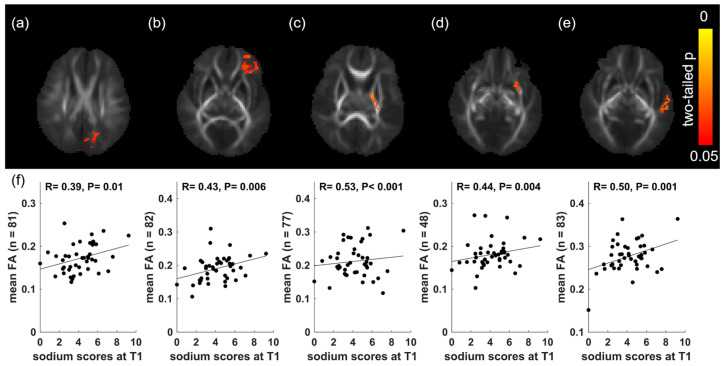
Clusters with positive correlations (two-tailed *p* ≤ 0.05, FWE corrected, cluster size ≥ 40 voxel size) between HEI-2015 sodium scores in the first trimester and neonatal brain FA values were identified using TFCE. The clusters are located in the parietal white matter (**a**), the anterior corona radiata (**b**), the posterior limb of the internal capsule (**c**), the external capsule (**d**), and temporal white matter (**e**) of the left hemisphere. Scatter plots showing a significant correlation (*p* ≤ 0.05) between the HEI-2015 sodium scores in the first trimester and the mean FA values of each cluster are in (**f**).

For the TBSS analyses of maternal HEI parameters and DTI-measured neonatal FA values, positive correlations (two-tailed *p* ≤ 0.05, FWE corrected) were identified in multiple clusters (with size ≥ 40 voxel size) in the brain WM using the TFCE at the voxel level. Specifically, positive correlations were found between sodium scores in the first trimester and FA values for clusters in the left parietal white matter (Figure 2a), left anterior corona radiata (Figure 2b), and in the posterior limb of the internal capsule, external capsule, and temporal white matter (Figure 2c–e) of the left hemisphere. No other clusters representing significant correlations between HEI scores and neonatal FA values were identified. Post hoc analyses were performed, and the relationships between HEI scores and mean FA values for each identified cluster were tested, with the newborn’s sex, postmenstrual age, and maternal BMI controlled. Figure 2f shows the scatter plots (mean FA values in the cluster versus HEI scores) for all subjects for the clusters identified in the TBSS analysis, and partial correlation coefficients as well as significance levels are included. Specifically, the sodium scores in the first trimester correlated with mean FA values in cluster a in left parietal white matter (R = 0.39, *p* = 0.01), in cluster b in left anterior corona radiata (R = 0.43, *p* = 0.006), in cluster c in left posterior limb of internal capsule (R = 0.53, *p* < 0.001), in cluster d in left external capsule (R = 0.44, *p* = 0.004), and in cluster e in left temporal white matter (R = 0.50, *p* = 0.001). 

## 4. Discussion

We studied maternal dietary quality assessed by HEI-2015 scores in healthy pregnant women at each trimester and evaluated their associations with their offspring’s brain white matter microstructural development at ~2 weeks of age. The FA values of the neonatal brain in multiple tracts were positively correlated with the mother’s sodium score in the first trimester. Our study is the first to link sodium intake patterns during pregnancy with changes in fetal brain development that may potentially impact neurodevelopment in later life. Our results indicate that a sodium intake pattern better aligned with the DGA 2015–2020 at the first trimester may positively influence neonatal WM microstructural development since FA is known to be a sensitive reflection of WM microstructural integrity and increases in FA values are associated with better WM development (such as more myelination) in the developing brain [30,31,32].

The HEI-2015 sodium scores in our study were 4.2 ± 1.8, 4.1 ± 1.8, and 4.5 ± 2.1 for the first, second, and third trimesters, respectively, which are similar to the data from NHANES cycle 2017–2018, which has a mean of 4.2 [33]. Sodium ubiquitously exists in many foods and beverages, and higher sodium intake is considered a contributing factor to high rates of high blood pressure, heart attack, and stroke. On the other hand, additional dietary sodium may be provided to preterm infants to improve postnatal growth, given the differences in how preterm-born and term-born may respond to sodium [34]. However, evidence linking supplemental sodium intake and preterm children’s neurodevelopmental outcomes is limited and inconclusive. For example, children born prematurely and supplemented to more sodium intakes for days 4–14 of postnatal life showed improved neurodevelopmental performance at 10–13 years of age [35], while a peak serum sodium of ≥150 mmol/L was shown to be associated with poor cognitive outcomes in preterm-born infants [36]. Moreover, few studies have examined the influence of maternal sodium intake during pregnancy on offspring brain growth and development, given the fact that in the fetus, sodium (Na) can be transferred via the syncytiotrophoblast Na+/K+-ATPase of the placenta [37]. As our study is the first to link sodium intake patterns during pregnancy with changes in fetal brain development, the underlying mechanism of our finding remains unclear, but studies in experimental models suggest some potential pathways and mechanisms of action on how sodium intake may affect the brain. One study in mice reported that excessive dietary sodium suppressed resting cerebral blood flow and endothelial function via circulating interleukin-17 that inhibited phosphorylation of endothelial nitric oxide synthase and reduced nitric oxide production in cerebral endothelial cells, leading to cognitive impairment which involved memory and learning skills [38]. The same research group also detected that high levels of sodium-induced hyperphosphorylation of tau protein in mice were followed by cognitive dysfunction [39]. In addition, another study showed the possible mechanism of effects of sodium associated with the down-regulation of synapse-related proteins, such as decreased phosphorylation of Ca2+/calmodulin-dependent protein kinase II (CaMKII) and postsynaptic density protein 95 (PSD95) expression in prefrontal and hippocampus of mice brain, resulting in impaired social behavior and object recognition memory dysfunction [40]. Of note, excessive sodium intake may also cause cognitive dysfunction by eliciting a neuroinflammatory environment [41,42,43] and triggering apoptosis in the brain of mice [42]. Furthermore, one study in rats found that offspring protein levels of myelin basic protein, calmodulin/CaMKII, and brain-derived neurotrophic factor were decreased or aberrantly expressed in the cerebral cortex and hippocampus due to maternal excessive sodium intake [44], suggesting that offspring brain responded to sodium in the same way as the brain within the same individual. Additionally, a study on mice reported that the offspring of maternal high-sodium diet subjects exhibited short- and long-term memory deficits [45]. The cognitive functions impaired by high sodium intake in the aforementioned animal studies are consistent with functions controlled by the WM tracts, for which we identified correlations between maternal sodium intake and neonatal WM microstructural development. For example, many studies reported memory association with the anterior corona radiate [46,47], parietal white matter [48], posterior limb of internal capsule [49,50,51], external capsule [52,53], and temporal white matter [54]. 

It is also not clear why only the sodium score during the first trimester of pregnancy was found to be associated with neonatal brain development. This may be associated with our relatively small sample size and inadequate power to identify relationships with sodium intake patterns later in pregnancy. Still, it may also suggest an early impact of sodium on the developing brain. In the first trimester, WM tracts start to develop, including the corpus callosum, the fornix, the anterior commissure, and the uncinate fasciculus [55]. The first trimester may be a critical period when the entire brain developmental process is vulnerable, and a disruption of the fetal environment may not only impact brain regions that are developing fast during the first trimester but also program effects on other brain regions yet to be developed. Monitoring of dietary intake and dietary quality starting from the first trimester and throughout the whole pregnancy can allow us to explore the timing and origin of the impact of different nutrition factors. Future studies with larger sample sizes and similar study designs will be important to provide more insight into this.

The total HEI-2015 scores for the pregnancy cohort in our study were 49.2 ± 7.2, 48.1 ± 10.1, and 49.2 ± 8.5 for the first, second, and third trimesters, respectively, which are lower than the data from NHANES cycle 2013–2018 that reported a mean of 53 for non-pregnant, non-lactating women, and 63 for pregnant women, respectively [56]. Among all the components we studied, intake of total protein foods was the most aligned with the DGA 2015–2020, while total fruit, greens and beans, refined grains, sodium, and added sugar were not well-aligned with the DGA 2015–2020. The reason that our data deviated slightly from the NHANES DATA may be partially attributed to regional effects, as data from a large cohort from rural areas showed a similar dietary pattern as our cohort, where health literacy may play a role in compliance to DGA [57]. In addition, adherence to the DGA may be impacted by demographic background and socioeconomic status [58].

Limitations of our study include the following: the relatively small sample size limited our capability of controlling more potential confounders; multiple comparison correction was conducted to account for multiple imaging measures in the voxel-wise analysis but not for multiple HEI parameters because of the exploratory nature of our report; MRI scans were conducted on a 1.5T scanner with relatively large voxel size, and a basic (instead of advanced) DTI protocol; and the HEI scores (account for energy intake per 1000 calories) might be skewed instead of using real nutrient intake quantity. Despite these limitations, using a 3-day food record, which is regarded as a more reliable measure of diet [59], and assessments repeated at each trimester during the pregnancy are both strengths for our study. We also performed brain imaging soon after birth, which is a time point that all prenatal influences have concluded while postnatal influences have mostly not started. 

## 5. Conclusions

Our results showed that the sodium intake pattern at the first trimester of pregnancy better aligned with the DGA 2015–2020 and was associated with better neonatal WM microstructural development measured by diffusion tensor imaging and provided novel data that can indicate the possible impact of maternal sodium intake patterns during early pregnancy on offspring brain development. 

## Figures and Tables

**Table 1:** HEI-2015 components, score range, and standards for scoring.

Component	Range	Scoring Standard	
		Minimum Score	Maximum Score
Total fruits	[0, 5]	No fruit	≥0.8 cup equivalents/1000 kcal
Whole fruits	[0, 5]	No whole fruit	≥0.4 cup equivalents/1000 kcal
Total vegetables	[0, 5]	No vegetables	≥1.1 cup equivalents/1000 kcal
Greens and beans	[0, 5]	No dark green vegetables or beans and peas	≥0.2 cup equivalents/1000 kcal
Whole grains	[0, 10]	No whole grains	≥1.5 oz equivalents/1000 kcal
Dairy	[0, 10]	No dairy	≥1.3 cup equivalents/1000 kcal
Total protein foods	[0, 5]	No protein foods	≥2.5 oz equivalents/1000 kcal
Seafood and plant proteins	[0, 5]	No seafood or plant proteins	≥0.8 oz equivalents/1000 kcal
Fatty acids *	[0, 10]	(PUFAs + MUFAs)/SFAs ≤1.2	(PUFAs + MUFAs)/SFAs ≥ 2.5
Refined grains	[0, 10]	≥4.3 oz equivalents/1000 kcal	≤1.8 oz equivalents/1000 kcal
Sodium	[0, 10]	≥2.0 g/1000 kcal	≤1.1 g/1000 kcal
Saturated fats	[0, 10]	≥16% of energy	≤8% of energy
Added sugars	[0, 10]	≥26% of energy	≤6.5% of energy

* PUFAs = polyunsaturated fatty acids; MUFAs = monounsaturated fatty acids; SFAs = saturated fatty acids.

**Table 2:** Demographic information of the study participants.

	Mean ± SD (or Counts)	Range (If Applicable)
Maternal age at delivery (years)	29.4 ± 4.0	[22.1, 38.2]
Maternal BMI at time of enrollment	26.2 ± 5.6	[18.3, 36.5]
Child sex (boys/girls)	23/21	
Age at MRI (days)	14.3 ± 1.6	[11, 19]
Gestational age at birth (weeks)	39.3 ± 1.0	[37.3, 40.7]
Postmenstrual age at MRI (days)	289.3 ± 6.5	[277, 300]
Birth weight (kg)	3.5 ± 0.5	[2.2, 4.6]
Birth length (cm)	50.6 ± 2.7	[43.2, 54.6]

**Table 3:** Descriptive statistics of HEI-2015 scores at first (T1), second (T2), and third (T3) trimesters.

	T1 (N = 43)		T2 (N = 44)		T3 (N = 44)	
	Mean ± Std	Range	Mean ± Std	Range	Mean ± Std	Range
Total fruits	2.2 ± 1.2	[0.0, 5.0]	2.1 ± 1.2	[0.0, 5.0]	2.2 ± 1.2	[0.1, 4.9]
Whole fruits	2.2 ± 1.4	[0.0, 4.9]	2.5 ± 1.3	[0.0, 5.0]	2.5 ± 1.5	[0.0, 5.0]
Total vegetables	3.1 ± 0.8	[1.5, 5.0]	3.0 ± 1.0	[0.9, 4.7]	2.9 ± 1.2	[0.6, 5.0]
Greens and beans	2.0 ± 1.0	[0.0, 4.2]	1.8 ± 1.4	[0.0, 4.7]	1.8 ± 1.3	[0.0, 5.0]
Whole grains	5.0 ± 2.2	[0.7, 9.1]	4.9 ± 2.8	[0.0, 10.0]	4.9 ± 2.6	[0.2, 9.9]
Dairy	5.9 ± 1.8	[0.8, 9.6]	6.1 ± 1.9	[2.3, 9.9]	5.6 ± 2.2	[2.0, 10.0]
Total protein foods	4.1 ± 0.7	[1.5, 5.0]	4.0 ± 0.9	[1.7, 5.0]	4.0 ± 0.9	[1.0, 5.0]
Seafood and plant proteins	2.3 ± 1.3	[0.0, 5.0]	2.2 ± 1.5	[0.0, 5.0]	2.4 ± 1.4	[0.0, 5.0]
Fatty acids	5.0 ± 1.7	[1.5, 8.9]	4.4 ± 1.9	[0.3, 8.5]	5.3 ± 2.1	[1.1, 9.8]
Refined grains	3.7 ± 2.4	[0.0, 9.7]	4.0 ± 2.7	[0.0, 10.0]	4.1 ± 2.6	[0.0, 10.0]
Sodium	4.2 ± 1.8	[0.0, 9.2]	4.1 ± 1.8	[0.9, 7.7]	4.5 ± 2.1	[0.0, 10.0]
Saturated fats	4.8 ± 1.7	[0.7, 8.2]	4.8 ± 1.6	[0.6, 8.4]	5.0 ± 1.7	[1.1, 9.0]
Added sugars	4.6 ± 1.3	[1.9, 7.7]	4.3 ± 1.6	[0.0, 7.9]	4.1 ± 1.8	[0.0, 7.1]
Total score	49.2 ± 7.2	[35.3, 63.6]	48.1 ± 10.1	[28.3, 68.7]	49.2 ± 8.5	[34.8, 66.8]

## Data Availability

The data presented in this study are available on request from the corresponding author. The data are not publicly available due to privacy.
